# Supplementary material for: Liver proteome alterations in psychologically distressed rats and a nootropic drug
Source: PeerJ. 2021 May 19;9:e11483. doi: 10.7717/peerj.11483 (PMC8140599; doi:10.7717/peerj.11483)
Supplement: Supplemental Information 1 — A detailed description of the in-gel digestion, the nanoLC-MS/MS analysis, and the protein search parameters are included. [file peerj-09-11483-s001.pdf]

Supplemental Methodology

In gel-digestion and nanoLC-MS/MS analysis

An in-gel protein digestion and a nanoLC-MS/MS of proteins analysis were performed according to the methodology previously described (Espinosa-Gómez et al. 2020). Each gel section was distained with 2.5 mM ammonium bicarbonate (NH_4HCO_3) in 50 % acetonitrile (ACN), and then dehydrated with 100 mL of 100 % ACN. Proteins in gel sections were reduced with 20 mL of 10 mM DTT in 50 mM NH_4HCO_3 and incubated for 45 min at 56 °C. Later, the samples were chilled at room temperature, alkylated by adding 20 µL of 100 mM iodoacetamide in 50 mM NH_4HCO_3 and incubated in the dark for 30 min. Next, gel cubes were firstly washed with 100 mL of 100 % ACN, secondly washed with 100 µL of 5 mM NH_4HCO_3, thirdly washed again with 100 mL of 100 % ACN, and finally dried using a CentriVap (Labconco Kansas, Missouri), each step for 5 min. The gels sections were rehydrated with 10 µL of digestion solution containing 12.5 ng/µL mass spectrometry grade Trypsin Gold (Promega, Madison, WI, USA) in 5 mM NH_4HCO_3 and incubated in a water bath at 37 °C overnight and the reaction stopped at –80 °C. The resulted peptides were extracted trice with 30 µL of 50 % ACN with 5 % formic acid by centrifugation at 1,000 x *g* for 30 s. Finally, samples were desalted with ZipTip-µC18 tips (Merck Millipore, Darmstadt, Germany) and dried using a CentriVap (Labconco Kansas, Missouri).
Peptide samples were reconstituted with 5 µL of 0.1 % formic acid were injected into a nanoViper™ C18 Nano-Trap column (3 μm, 75 μm X 2 cm, Dionex™) at 3 µL min^{-1} flow rate, and then separated on an EASY spray C18 RSLC column (2 μm, 75 μm x 25 cm) with a flow rate of 300 nL·min^{-1} connected to an UltiMate 3000 RSnanoLC system (Thermo-Fisher Scientific, San Jose, CA), and interfaced with an Orbitrap Fusion™ Tribid™ (Thermo-Fisher Scientific, San Jose, CA) mass spectrometer equipped with an "EASY Spray" nano ion source (Thermo-Fisher Scientific, San Jose, CA). For peptide separation, a chromatographic gradient using MS grade water with 0.1 % formic acid (solvent A) and 0.1 % formic acid in 90 % ACN (solvent B) for 30 min was set as followed: 10 min solvent A, 5–25 % solvent B within 20 min, 25–95 % solvent B for 5 min, 95 % solvent B for 10 min and 95–5 % solvent B for 5 min. The mass spectrometer was operated in positive ion mode with nanospray voltage set at 3.5 kV and source temperature set at 280 °C. External calibrant included caffeine, Met-Arg-Phe-Ala (MRFA), and Ultramark™ 1621.
The mass spectrometer was operated in a data-dependent mode. Briefly, survey full-scan MS spectra were acquired in the Orbitrap analyzer, scanning of the mass range was set to 350–1,500 m/z at a resolution of 120,000 (FWHM) using an automatic gain control (AGC) setting to 4.0e5 ions, maximum injection time to 50 ms, dynamic exclusion 1 at the 90S and 10 ppm mass tolerance. Subsequently, a top speed survey scan for 3 s was selected for subsequent decision tree-based Orbitrap collision-induced dissociation (CID) or higher-energy collisional dissociation (HCD) fragmentation (Griep-Raming et al. 2011; Swaney, McAlister, and Coon 2008). The signal threshold for triggering an MS/MS event was set to 1.0e4 and the normalized collision energy was set to 35 % and 30 % for CID and HCD, respectively. The AGC of 3.0e4 and isolation window of 1.6 m/z was set for both fragmentations. An additional parameter for CID included activation Q was set to 0.25 ms and injection time to 50 ms. For HCD, the first mass was set to 120 m/z and injection time to 100 ms. The settings for the decision tree were as follows: for HCD fragmentation charge states 2 or 3 were scan in a range of 650-1200 m/z, charge states 4 were scan in a range of 900-1200 m/z, and charge

states 5 were scan in a range of 950-1200 m/z; for CID fragmentation charge states 3 were scan in a range of 650-1200 m/z, charge state 4 were scan in a range of 300-900 m/z, and charge state 5 in scan range of 300-950 m/z. All data were acquired with Xcalibur 4.0.27.10 software (Thermo-Fisher Scientific) (Espinosa-Gómez et al. 2020).

Protein search parameters used in data analysis

The parameters of the analysis comprised: full-tryptic protease specificity, two missed cleavage allowed, static modifications covered carbamidomethylation of cysteine (+57.021 Da). Furthermore, dynamic modifications included methionine oxidation (+15.995 Da) and deamidation in asparagine/glutamine (+0.984 Da). For the MS2 method, in which identification was performed at high resolution in the Orbitrap, precursor and fragment ion tolerances of ±10 ppm and ±0.2 Da were applied. The resulting peptide hits were filtered for a maximum of 1 % FDR using the Percolator algorithm (Käll et al. 2007).
