# Supplementary figures and images for: Liver proteome alterations in psychologically distressed rats and a nootropic drug
Source: PeerJ. 2021 May 19;9:e11483. doi: 10.7717/peerj.11483 (PMC8140599; doi:10.7717/peerj.11483)

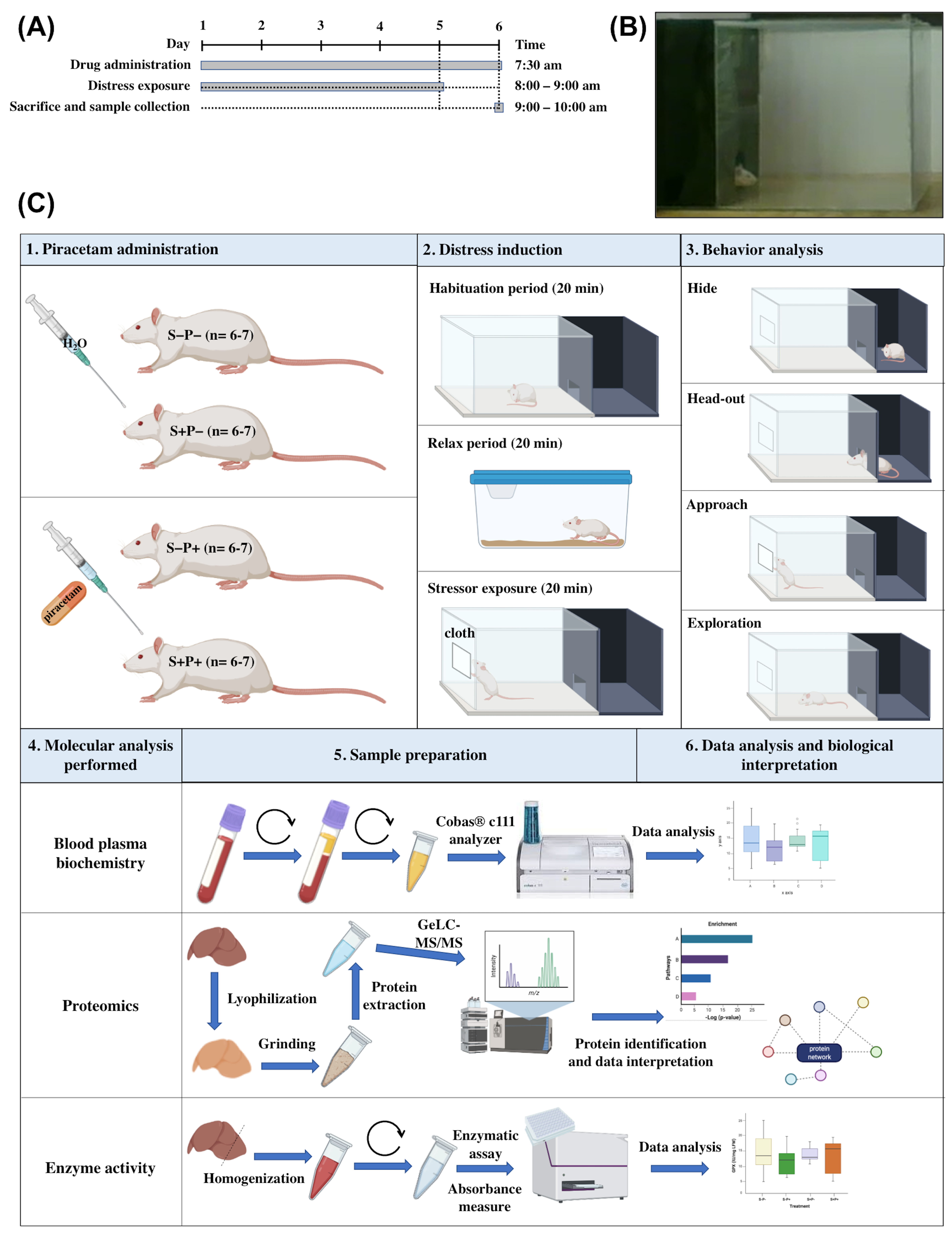

Supplement: Supplemental Information 2 — For six consecutive days, rats received piracetam (600 mg/kg), dispensed orally using a cannula, in single dose, at 7:30 am. From the first day until the fifth day, the animals were exposed to the stressor (at 8 am, after drug administration). In the sixth day, rats were sacrificed. [file peerj-09-11483-s002.png]

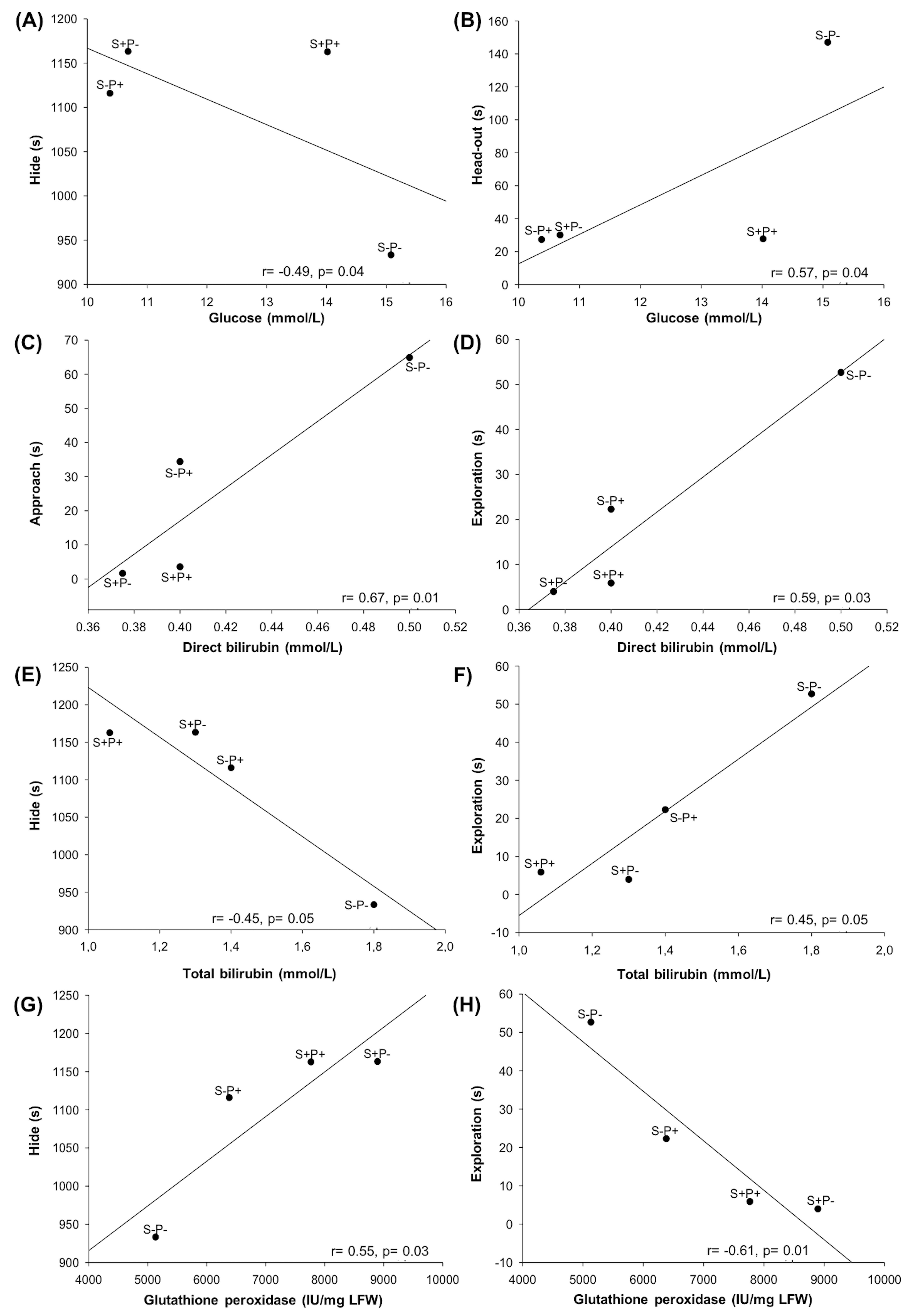

Supplement: Supplemental Information 3 — The glucose level was negatively correlated with the time the rat spent hiding (A) and positively with the head-out position (B). The direct bilirubin level was positively correlated with the time the rat spent approaching (C) and exploring (D). The total bilirubin level was negatively correlated with the time the rat spent hiding (E) and positively with the exploring time (F). The GPX activity was positively correlated to the time the rat spent hiding (G) and negatively with the exploring time (H). [file peerj-09-11483-s003.png]
