# Supplementary material for: Liver proteome alterations in psychologically distressed rats and a nootropic drug
Source: PeerJ. 2021 May 19;9:e11483. doi: 10.7717/peerj.11483 (PMC8140599; doi:10.7717/peerj.11483)
Supplement: Supplemental Information 4 — S−P−: unique protein detected in S−P − condition; S+P−: unique protein detected in S+P− condition; S+P+: unique protein detected in S+P+ condition. [file peerj-09-11483-s004.docx]

|  |  |  | Fold change | | |
| --- | --- | --- | --- | --- | --- |
| Uniprot ID | **Protein name (gen symbol)** | | **S+P−/S−P−** | **S+P+/S+P−** | **S−P+/ S−P−** |
| P04762 | Catalase (Cat) | | 1.54 | 1.18 | 1.42 |
| D4ADD7 | Glutaredoxin 5 (Glrx5) | | S+P− | 0.44 | S−P− |
| P04041 | Glutathione peroxidase 1 (Gpx1) | | 1.0 | 1.75 | 1.21 |
| P00502 | Glutathione S-transferase alpha-1 (Gsta1) | | 0.82 | 1.22 | 0.66 |
| P04903 | Glutathione S-transferase alpha-2 (Gsta2) | | 0.82 | 1.22 | 0.66 |
| P04904 | Glutathione S-transferase alpha-3 (Gsta3) | | 0.29 | 1.72 | 0.50 |
| P14942 | Glutathione S-transferase alpha-4 (Gsta4) | | 0.46 | 2.19 | 1.00 |
| P24473 | Glutathione S-transferase kappa 1 (Gstk1) | | 1.60 | 2.44 | 1.60 |
| P04905 | Glutathione S-transferase Mu (Gstm1) | | 0.83 | 1.98 | 1.19 |
| P08009 | Glutathione S-transferase Mu 7 (Gstm7) | | S−P− | S−P− | S−P− |
| P30713 | Glutathione S-transferase theta-2 (Gstt2) | | 3.56 | 2.00 | 5.20 |
| P08009 | Glutathione S-transferase Yb-3 (Gstm3) | | S+P- | 0.62 | S−P+ |
| P46413 | Glutathione synthetase (Gss) | | 0.31 | 1.00 | 2.25 |
| P08011 | Microsomal glutathione S-transferase 1 (Mgst1) | | 7.0 | 23.1 | S−P− |
| A0A0G2JU12 | Microsomal glutathione S-transferase 2 (Mgst2) | | S+P− | S+P+ | n.d. |
| D4ADS4 | Microsomal glutathione S-transferase 3 (Mgst3) | | S+P− | 1.00 | S−P+ |
| G3V7I0 | Peroxiredoxin 3 (Prdx3) | | 1.00 | 1.00 | 1.63 |
| Q9R063 | Peroxiredoxin 5, isoform CRA_c (Prdx5) | | 0.55 | 1.81 | 1.28 |
| P35704 | Peroxiredoxin-2 (Prdx2) | | 1.00 | 1.52 | 1.52 |
| O35244 | Peroxiredoxin-6 (Prdx6) | | 0.83 | 2.42 | 0.56 |
| P07632 | Superoxide dismutase [Cu-Zn] (Sod1) | | 0.48 | 0.60 | 0.72 |
| O89049 | Thioredoxin reductase 1, cytoplasmic (Txnrd1) | | 1.56 | 1.39 | 2.17 |
