# Supplementary material for: Liver proteome alterations in psychologically distressed rats and a nootropic drug
Source: PeerJ. 2021 May 19;9:e11483. doi: 10.7717/peerj.11483 (PMC8140599; doi:10.7717/peerj.11483)
Supplement: Supplemental Information 5 — The fold change was obtained from the ratio between the relative protein abundance based on the emPAI value of the indicated groups. S−P−, S+P−, S+P+, S −P+ indicate proteins that were detected only in S −P−, S+P−, S+P+, or S−P+ group respectively. n.d. indicates protein that was not detected in the experimental group. PD and both indicate proteins that are categorized only in PD pathway or in both pathways, respectively. S−P −, rats neither exposed to stress nor with piracetam treatment; S−P+, rats not exposed to stress but treated with piracetam; S+P−, rats exposed to stress without piracetam treatment; S+P+, rats exposed to stress and treated with piracetam. [file peerj-09-11483-s005.docx]

| Uniprot ID | Protein name (gene symbol) | Pathway involved | Fold change | | |
| --- | --- | --- | --- | --- | --- |
|  |  |  | S+P−/S−P− | S+P+/S+P− | S−P+/S−P− |
| Oxidative Phosphorylation | | | | | |
| *NADH dehydrogenase (Complex I)* | | | | | |
| B2RZD6 | NDUFA4, mitochondrial complex associated (Ndufa4) | both | 2.47 | 1.00 | 2.47 |
| Q5XIH3 | NADH dehydrogenase [ubiquinone] flavoprotein 1, mitochondrial (Ndufv1) | both | n.d. | n.d. | S−P+ |
| A0A0G2JVL6 | NADH dehydrogenase [ubiquinone] 1 alpha subcomplex subunit 8 (Ndufa8) | both | S−P− | S+P+ | 2.26 |
| Q5BK63 | NADH dehydrogenase [ubiquinone] 1 alpha subcomplex subunit 9, mitochondrial | both | 1.39 | 1.31 | 1.82 |
| Q561S0 | NADH dehydrogenase [ubiquinone] 1 alpha subcomplex subunit 10, mitochondrial (Ndufa10) | both | S−P− | n.d. | 0.71 |
| D3ZE15 | NADH dehydrogenase [ubiquinone] 1 alpha subcomplex subunit 13-like (Ndufa13) | both | 0.70 | 1.43 | 0.70 |
| D3ZF13 | Acyl carrier protein (Ndufab1) | both | S+P− | S+P− | n.d. |
| D4A565 | NADH dehydrogenase (Ubiquinone) 1 beta subcomplex, 5 (Predicted), isoform CRA_b (Ndufb5) | both | 1.68 | 1.00 | 0.45 |
| D3ZZ21 | NADH dehydrogenase (Ubiquinone) 1 beta subcomplex, 6 (Predicted) (Ndufb6) | both | 2.78 | 1.49 | 0.43 |
| Q66HF1 | NADH-ubiquinone oxidoreductase 75 kDa subunit, mitochondrial (Ndufs1) | both | 0.30 | 1.00 | 0.63 |
| D3ZG43 | NADH dehydrogenase (Ubiquinone) Fe-S protein 3 (Predicted), isoform CRA_c (Ndufs3) | both | 1.90 | 1.28 | 1.42 |
| P19234 | NADH dehydrogenase [ubiquinone] flavoprotein 2, mitochondrial (Ndufv2) | both | 0.70 | 1.00 | 1.34 |
| *Succinate dehydrogenase (Complex II)* | | | | | |
| Q920L2 | Succinate dehydrogenase [ubiquinone] flavoprotein subunit, mitochondrial (Sdha) | both | 1.93 | 0.67 | 1.00 |
| P21913 | Succinate dehydrogenase [ubiquinone] iron-sulfur subunit, mitochondrial (Sdhb) | both | 1.00 | 1.84 | 1.00 |
| *Cytochrome b-c1 complex (Complex III)* | | | | | |
| Q68FY0 | Cytochrome b-c1 complex subunit 1, mitochondrial (Uqcrc1) | PD | 1.00 | 0.47 | 1.58 |
| P20788 | Cytochrome b-c1 complex subunit Rieske, mitochondrial (Uqcrfs1) | both | 0.29 | 2.14 | 0.62 |
| *Cytochrome c oxidase (Complex IV)* | | | | | |
| P00406 | Cytochrome c oxidase subunit 2 (Cox2) | both | 0.65 | 1.55 | 0.65 |
| P11240 | Cytochrome c oxidase subunit 5A, mitochondrial (Cox5a) | both | 1.52 | 0.66 | 0.59 |
| D3ZD09 | Cytochrome c oxidase subunit 6B1 (Cox6b1) | both | 2.39 | 1.00 | 2.39 |
| *ATP synthase (Complex V)* | | | | | |
| P35435 | ATP synthase subunit gamma, mitochondrial (Atp5f1c) | PD | 0.47 | S+P− | S−P− |
| D3ZAF6 | ATP synthase subunit f, mitochondrial (Atp5mf) | PD | 6.62 | 1.00 | 1.00 |
| P19511 | ATP synthase F(0) complex subunit B1, mitochondrial (Atp5pb) | PD | 2.15 | 1.60 | 3.44 |
| ADP/ATP translocases | | | | | |
| Q05962 | ADP/ATP translocase 1 (Slc25a4) | PD | S−P− | n.d. | S−P− |
| Q09073 | ADP/ATP translocase 2 (Slc25a5) | PD | S+P− | 0.63 | S-P+ |
| Protein transport via vesicles | | | | | |
| Q66HM2 | Adaptor protein complex 2 subunit alpha-2 (Ap2a2) | PD | S+P− | 1.52 | S−P+ |
| P62944 | Adaptor protein complex 2 complex subunit beta (Ap2b1) | PD | S+P− | 1.00 | n.d. |
| Regulation of cytoskeleton organization | | | | | |
| Q8CFN2 | Cell division control protein 42 homolog (Cdc42) | both | 0.59 | 1.00 | 0.59 |
| Fatty acid metabolism | | | | | |
| P05182 | Cytochrome P450 2E1 (Cyp2e1) | both | 3.20 | 0.65 | 2.08 |
| Response to oxidative stress | | | | | |
| P04041 | Glutathione peroxidase 1 (Gpx1) | PD | 1.00 | 1.75 | 1.21 |
| O88767 | Protein DJ-1 (Park7) | PD | 0.51 | 2.64 | 1.80 |
| P07632 | Superoxide dismutase [Cu-Zn] (Sod1) | PD | 0.48 | 0.60 | 0.72 |
| Apoptosis regulation | | | | | |
| D3ZFQ8 | Cytochrome c-1 (Cyc1) | both | 0.62 | 1.60 | 1.00 |
| A0A0G2K151 | Apolipoprotein E (Apoe) | PD | S−P− | n.d. | 0.63 |
| B0BNB9 | HtrA serine peptidase (Htra2) | PD | 1.57 | 0.64 | 1.57 |
| P29117 | Peptidyl-prolyl cis-trans isomerase F, mitochondrial (Ppif) | PD | 1.60 | 1.00 | 1.60 |
| F7EPH4 | Pyrophosphatase (inorganic) (Ppa1) | PD | 0.58 | 2.17 | 1.87 |
| Q9R1Z0 | Voltage-dependent anion-selective channel protein 3 (Vdac3) | PD | S+P− | S+P− | S−P+ |
| Glycolisis | | | | | |
| P12928 | Pyruvate kinase PKLR (Pklr) | both | 0.72 | 1.69 | 1.00 |
| Cellular energy metabolism regulation | | | | | |
| P80386 | 5'-AMP-activated protein kinase subunit beta-1 (Prkab1) | both | S−P− | S+P+ | 2.15 |
