# Supplementary material for: Liver proteome alterations in psychologically distressed rats and a nootropic drug
Source: PeerJ. 2021 May 19;9:e11483. doi: 10.7717/peerj.11483 (PMC8140599; doi:10.7717/peerj.11483)
Supplement: Supplemental Information 6 — Most abundant indicates proteins with fold change (FD) ≥1.5. Less abundant indicates proteins with FD ≤0.66. n.d. indicates protein that was not detected in the experimental group. [file peerj-09-11483-s006.docx]

| **Pathway** | **S+P− vs. S−P−** | **S+P+ vs. S+P−** | **S−P+ vs. S−P−** |
| --- | --- | --- | --- |
| **Protein processing in ER** | *Most abundant*  CANX, GANAB, HSP90AA1, HSP90AB1, HSPH1, HYOU1, RPN2, SAR1A, SEC13, TXNDC5, UBQLN1, UGGT1, VCP | *Most abundant*  CALR, ERP29, PDIA4, PDIA6, RAD23B, RPN1, SAR1A, SEC23A | *Most abundant*  n.d. |
|  | *Less abundant*  CALR, ERP29, PDIA6, RPN1, RAD23B, SAR1A, SAR1B, SEC24A | *Less abundant*  n.d. | *Less abundant*  n.d. |
| **Proteasome** | *Most abundant*  PSMA4, PSMB4, PSMC1, PSMC6, PSMD2, PSMD3, NEDD4, YWHAH, YWHAQ, YWHAZ, YWHAE, VIM | *Most abundant*  PSMA1, PSMA2, PSMA6, PSMA7, PSMB1, PSMC6, PSMD3, PSMD7, PSMD12 | *Most abundant*  n.d. |
|  | *Less abundant*  PSMA6, PSMB1, PSMB3, PSMC2, PSMC4, PSMD7, PSMD12, PSMD14, PSME1, PSME2 | *Less abundant*  PSMA8, PSMB4, PSMC1, PSMC3, PSMC4, PSME2 | *Less abundant*  PSMA6, PSMC3, PSMC4, PSMD12, PSMD14, PSME1, PSME2 |
| **Metabolism of xenobiotics by Cyt P450** | *Most abundant*  ADH1, ADH4, AKR7A3, CYP2E1, EPHX1, GSTK1, GSTM2, GSTT2, SULT2A1, MGST1, MGST3, MGST2, UGT2B1, UGT2B35 | *Most abundant* GSTA1, GSTA4, GSTM1, GSTK1, GSTT2, MGST1, RGD1559459, UGT1A2, UGT2B35 | *Most abundant*  ADH4, AKR7A2, AKR7A3, CYP2E1, EPHX1, GSTK1, GSTM2, GSTT2, MGST1, MGST3, RGD1559459, UGT1A5, UGT1A6, UGT2B, UGT2B35, UGT2B37 |
|  | *Less abundant*  GSTA1, GSTA4, GSTM7, RGD1559459, UGT1A1, UGT1A5, UGT2B1 | *Less abundant*  ADH4, AKR7A2, CYP2E1, GSTM2, MGST2, SULT2A1, UGT1A5, UGT2B10, UGT2B35 | *Less abundant*  GSTA1, GSTA2, GSTA5, GSTM7, SULT2A1, UGT2B1, UGT2B35 |
