# Supplementary material for: Liver proteome alterations in psychologically distressed rats and a nootropic drug
Source: PeerJ. 2021 May 19;9:e11483. doi: 10.7717/peerj.11483 (PMC8140599; doi:10.7717/peerj.11483)
Supplement: Supplemental Information 7 — Significant values are marked in bold (* p ≤ 0.05; †p ≤ 0.01). [file peerj-09-11483-s007.docx]

| Characterist | 1 | 2 | 3 | 4 | 5 | 6 | 7 | 8 | 9 | 10 | 11 | 12 | 13 | 14 | 15 | 16 | 17 | 18 | 19 | 20 | 21 | 22 | 23 | 24 | 25 |
| --- | --- | --- | --- | --- | --- | --- | --- | --- | --- | --- | --- | --- | --- | --- | --- | --- | --- | --- | --- | --- | --- | --- | --- | --- | --- |
| 1.Treatment | 1.00 |  |  |  |  |  |  |  |  |  |  |  |  |  |  |  |  |  |  |  |  |  |  |  |  |
| 2.Glucose | -0.10 | 1.00 |  |  |  |  |  |  |  |  |  |  |  |  |  |  |  |  |  |  |  |  |  |  |  |
| 3.Triglycerides | -0.09 | **0.66†** | 1.00 |  |  |  |  |  |  |  |  |  |  |  |  |  |  |  |  |  |  |  |  |  |  |
| 4.Cholesterol | -0.07 | 0.16 | -0.08 | 1.00 |  |  |  |  |  |  |  |  |  |  |  |  |  |  |  |  |  |  |  |  |  |
| 5.Direct bilirubin | -0.35 | 0.26 | 0.27 | 0.15 | 1.00 |  |  |  |  |  |  |  |  |  |  |  |  |  |  |  |  |  |  |  |  |
| 6.Indirect bilirubin | **-0.63*** | 0.36 | -0.01 | 0.53 | 0.31 | 1.00 |  |  |  |  |  |  |  |  |  |  |  |  |  |  |  |  |  |  |  |
| 7.Total bilirubin | -0.58 | 0.02 | -0.23 | 0.04 | 0.28 | **0.76†** | 1.00 |  |  |  |  |  |  |  |  |  |  |  |  |  |  |  |  |  |  |
| 8.AST | 0.23 | 0.14 | -0.02 | 0.30 | 0.00 | 0.52 | 0.34 | 1.00 |  |  |  |  |  |  |  |  |  |  |  |  |  |  |  |  |  |
| 9.ALT | 0.00 | 0.06 | 0.04 | 0.04 | 0.08 | 0.31 | 0.09 | 0.08 | 1.00 |  |  |  |  |  |  |  |  |  |  |  |  |  |  |  |  |
| 10.Albumin | -0.27 | 0.13 | -0.20 | 0.29 | -0.07 | 0.09 | -0.10 | -0.31 | -0.38 | 1.00 |  |  |  |  |  |  |  |  |  |  |  |  |  |  |  |
| 11.Total protein | -0.19 | 0.23 | 0.14 | **0.49*** | -0.28 | 0.28 | 0.06 | 0.30 | -0.39 | 0.13 | 1.00 |  |  |  |  |  |  |  |  |  |  |  |  |  |  |
| 12.Urea | -0.30 | -0.29 | -0.36 | 0.10 | -0.01 | 0.41 | 0.11 | -0.05 | 0.29 | 0.20 | -0.27 | 1.00 |  |  |  |  |  |  |  |  |  |  |  |  |  |
| 13.Creatinine | -0.19 | 0.40 | 0.13 | 0.24 | -0.23 | 0.29 | 0.24 | 0.22 | 0.11 | 0.28 | 0.42 | -0.03 | 1.00 |  |  |  |  |  |  |  |  |  |  |  |  |
| 14.BUN | -0.31 | -0.24 | -0.33 | 0.08 | -0.02 | 0.41 | 0.12 | -0.06 | 0.20 | 0.25 | -0.23 | **0.99†** | 0.01 | 1.00 |  |  |  |  |  |  |  |  |  |  |  |
| 15.Total SOD | 0.29 | -0.10 | 0.14 | -0.10 | 0.09 | -0.37 | -0.22 | 0.12 | 0.29 | -0.06 | -0.35 | -0.05 | -0.07 | -0.08 | 1.00 |  |  |  |  |  |  |  |  |  |  |
| 16.SOD2 | 0.18 | 0.47 | 0.14 | -0.04 | -0.19 | 0.14 | -0.03 | -0.07 | 0.27 | -0.11 | 0.02 | -0.33 | 0.40 | -0.30 | -0.43 | 1.00 |  |  |  |  |  |  |  |  |  |
| 17.SOD1 | 0.08 | -0.12 | 0.19 | 0.09 | 0.00 | -0.11 | -0.06 | 0.11 | 0.36 | 0.13 | -0.29 | 0.08 | 0.01 | 0.05 | **0.86†** | -0.28 | 1.00 |  |  |  |  |  |  |  |  |
| 18.CAT | -0.11 | 0.41 | 0.26 | 0.31 | 0.27 | 0.32 | 0.17 | 0.04 | -0.06 | 0.23 | 0.03 | 0.00 | 0.49 | 0.05 | -0.39 | **0.53*** | -0.23 | 1.00 |  |  |  |  |  |  |  |
| 19.GST | 0.33 | 0.35 | 0.05 | 0.01 | 0.38 | -0.11 | -0.23 | 0.09 | 0.19 | -0.04 | -0.05 | -0.45 | 0.07 | -0.46 | 0.15 | 0.35 | -0.06 | 0.12 | 1.00 |  |  |  |  |  |  |
| 20.GR | -0.31 | 0.02 | 0.17 | -0.09 | 0.12 | 0.31 | 0.15 | -0.03 | 0.41 | -0.20 | -0.04 | 0.20 | -0.07 | 0.19 | 0.01 | -0.08 | 0.12 | -0.28 | -0.42 | 1.00 |  |  |  |  |  |
| 21.GPX | **0.61*** | -0.02 | -0.04 | -0.11 | -0.52 | -0.21 | -0.31 | 0.31 | 0.25 | -0.21 | -0.13 | 0.04 | 0.20 | 0.01 | 0.34 | 0.19 | 0.26 | 0.13 | 0.18 | -0.47 | 1.00 |  |  |  |  |
| 22.Hide | **0.82†** | **-0.49*** | -0.38 | -0.01 | **-0.64*** | -0.48 | **-0.45*** | 0.26 | -0.04 | -0.26 | 0.00 | 0.00 | -0.25 | -0.05 | 0.26 | -0.13 | 0.17 | -0.43 | -0.01 | -0.19 | **0.55*** | 1.00 |  |  |  |
| 23.Exploration | **-0.83†** | 0.30 | 0.20 | -0.05 | **0.59*** | 0.43 | **0.45*** | -0.39 | 0.00 | 0.26 | -0.02 | -0.03 | 0.13 | 0.00 | -0.36 | 0.17 | -0.26 | 0.33 | 0.03 | 0.11 | **-0.61*** | **-0.95†** | 1.00 |  |  |
| 24.Head-out | **-0.73†** | **0.57*** | 0.32 | 0.04 | 0.54 | 0.48 | 0.43 | -0.17 | -0.01 | 0.32 | 0.01 | 0.01 | 0.34 | 0.10 | -0.23 | 0.22 | -0.14 | **0.50*** | 0.09 | 0.21 | -0.49 | **-0.93†** | **0.83†** | 1.00 |  |
| 25.Approach | **-0.72†** | 0.31 | 0.49 | -0.03 | **0.67*** | 0.39 | 0.35 | -0.24 | 0.14 | 0.04 | 0.01 | 0.00 | 0.08 | -0.02 | -0.16 | -0.14 | -0.12 | 0.15 | -0.16 | 0.16 | -0.47 | **-0.84†** | **0.83†** | **0.59†** | 1.00 |
